# Urban women's socioeconomic status, health service needs and utilization in the four weeks after postpartum hospital discharge: findings of a Canadian cross-sectional survey

**DOI:** 10.1186/1472-6963-8-203

**Published:** 2008-10-03

**Authors:** Christine Kurtz Landy, Wendy Sword, Donna Ciliska

**Affiliations:** 1 School of Nursing, McMaster University, 1200 Main Street W., Hamilton, Ontario, L8N 3Z5, Canada

## Abstract

**Background:**

Postpartum women who experience socioeconomic disadvantage are at higher risk for poor health outcomes than more advantaged postpartum women, and may benefit from access to community based postpartum health services. This study examined socioeconomically disadvantaged (SED) postpartum women's health, and health service needs and utilization patterns in the first four weeks post hospital discharge, and compared them to more socioeconomically advantaged (SEA) postpartum women's health, health service needs and utilization patterns.

**Methods:**

Data collected as part of a large Ontario cross-sectional mother-infant survey were analyzed. Women (N = 1000) who had uncomplicated vaginal births of single 'at-term' infants at four hospitals in two large southern Ontario, Canada cities were stratified into SED and SEA groups based on income, social support and a universally administered hospital postpartum risk screen. Participants completed a self-administered questionnaire before hospital discharge and a telephone interview four weeks after discharge. Main outcome measures were self-reported health status, symptoms of postpartum depression, postpartum service needs and health service use.

**Results:**

When compared to the SEA women, the SED women were more likely to be discharged from hospital within the first 24 hours after giving birth [OR 1.49, 95% CI (1.01–2.18)], less likely to report very good or excellent health [OR 0.48, 95% CI (0.35–0.67)], and had higher rates of symptoms of postpartum depression [OR 2.7, 95% CI(1.64–4.4)]. No differences were found between groups in relation to self reported need for and ability to access services for physical and mental health needs, or in use of physicians, walk-in clinics and emergency departments. The SED group were more likely to accept public health nurse home visits [OR 2.24, 95% CI(1.47–3.40)].

**Conclusion:**

Although SED women experienced poorer mental and overall health they reported similar health service needs and utilization patterns to more SEA women. The results can assist policy makers, health service planners and providers to develop and implement necessary and accessible services. Further research is needed to evaluate SED postpartum women's health service needs and barriers to service use.

## Background

Although socioeconomically disadvantaged (SED) populations can benefit from the use of health and social services [1], there is a paucity of published research regarding SED women's health and social service needs and utilization patterns during the postpartum period. Women who are socioeconomically disadvantaged often experience inequities in health and health care. In their daily lives they face chronic stressors such as poverty, lack of social support, isolation, racism, violence, language barriers, and low levels of education [2,3]. These challenges create a complex burden of psychosocial, functional, and physical health risks which can obstruct their access to material resources and health care, and in turn put them at high risk for poor health outcomes and poor quality of life [2-4]. During the postpartum period SED women experience higher rates of postpartum depression, with reported rates between 22% and 30% [5-8] compared to 13% overall prevalence in the general population [9,10]. They are more likely to suffer from iron deficiency than their more SEA counterparts [11]. In addition SED postpartum women have more problems adjusting to the parenting role [12], report higher rates of intimate partner violence [13,14], are less likely to breastfeed [15] and are less likely to have a partner [16].

Studies report that SED postpartum women may have shorter hospital stays and may not be getting appropriate follow-up in the community [17]. In the prenatal period, American and Canadian studies show that SED women do not use recommended levels of health care even when financial barriers are lifted [18-21]. This pattern of health service utilization may carry over into the postpartum period, and may be reinforced by the problem that postpartum health care is often neglected, especially as it pertains to women's health [22-24]. Yet postpartum care integrated into a prenatal-postnatal care continuum is likely just as important in promoting short and long-term health as prenatal care, particularly for SED women and, in turn, for their children [22,24,25].

In Ontario, Canada, 98% of women give birth in hospital [26]. Hospitalization and visits to health care providers (physicians, midwives and nurses practitioners) and home visits by public health nurses are paid for by public health insurance and government programs with no additional out of pocket costs to patients in accordance with the Canada Health Act [27,28]. Over the last two decades length of postpartum hospital stay has shortened to an average of two days for women who have vaginal births [29]. Women access most of their postpartum care in the community. In the late 1990s the Ontario government responded to health professionals' concerns about the safety of the shortened length of postpartum hospital stay and the lack of standardized community based postpartum care by introducing the Healthy Babies Healthy Children Postpartum Enhancement [28]. Under this program all women are to be offered a 60 hour postpartum hospital stay, a public health nurse telephone call within 48 hours after hospital discharge to assess postpartum adjustment and family's level of risk for poor health outcomes, and an offer of a public health nurse home visit in which postpartum adjustment, infant and maternal health, and family functioning would be assessed and families would be referred to appropriate community services [28]. Inherent in the program is the assumption that those most in need will use the services offered and that there is appropriate and responsive services available for all postpartum women and their infants. However there is a dearth of information about which health and social services SED postpartum women in fact need and use to help them through the often challenging physical, emotional, and social changes that characterize the postpartum period.

This manuscript presents the findings of Phase 1 of a mixed methods study (undertaken from 2003 to 2005) examining health and social service needs and utilization patterns of SED postpartum women in the first four weeks after hospital discharge. The purpose of this phase of the study is to describe SED women's postpartum health at four weeks following hospital discharge and to compare it to more socioeconomically advantaged (SEA) postpartum women. In addition the SED women's postpartum health service needs and utilization of needed services will be described and compared to those of more SEA women.

## Methods

Data collected from postpartum women recruited at the four large urban hospital sites of The Ontario Mother Infant Survey II, a multi-site cross-sectional survey, in Ontario, Canada are presented in this paper. The Ontario Mother Infant Survey II was conducted to evaluate health outcomes, and health and social service needs and utilization patterns, and costs of care for postpartum women and their newborns in the first four weeks following hospital discharge. The focus of this larger study was not on socioeconomically disadvantaged postpartum women. However the research produced some important findings about this group that have not been reported in the literature. Ethical approval was received from the four hospital sites and McMaster University's research ethics board.

Trained research assistants on the postpartum units at the four hospital sites approached eligible postpartum women and explained the study. Women were eligible if they gave vaginal birth to full term singleton infants, were discharged together with their infant, were able to communicate in one of the five study languages (English, French, Spanish, Mandarin and Cantonese) and had a telephone. The first 250 postpartum women who were eligible and gave informed consent at each site were recruited into the study. In total 1000 women were recruited at the four sites.

Participants completed a self-administered questionnaire collecting their sociodemographic information before postpartum hospital discharge. In addition, for the purpose of this study, the research assistants collected the participants' Parkyn Postpartum Screen scores from their hospital charts. The Parkyn Postpartum Screen is a multidimensional tool assessing family socioeconomic and health risk, universally administered in Ontario postpartum hospital units to screen for families at high risk for poor health outcomes [30]. A score of 9 or above is indicative of high risk for poor health outcomes. Four weeks after hospital discharge participants undertook a structured telephone interview administered by trained interviewers. The interview included questions about hospital length of stay, maternal and infant health, social support, needs for care, ability to access care, and health and social services utilization. The Duke-UNC Functional Social Support Questionnaire [31], the Edinburgh Postnatal Depression Scale (EPDS) [32], and a modified Health and Social Service Utilization Questionnaire [33] were used in the telephone interviews to examine social support, risk of postpartum depression, and participants' recall of service use respectively. The Duke-UNC Functional Social Support Questionnaire contains two subscales measuring affective and confidant support. The subscales are reliable with reported alpha coefficients for confidant support = 0.62 and for affective support = 0.76 [31]. The EPDS is a valid measure of risk for postpartum depression with the following psychometric properties: sensitivity 86%, specificity 78%, positive predictive value 73%, split-half reliability 0.88, and alpha coefficient 0.87 [32]. Women scoring 12 or above on the EPDS were considered at high risk for postpartum depression. The Health and Social Service Utilization Questionnaire has adequate levels of agreement with clinic records (between 0.72 and 0.99 and Kappa ranges from 0.48 to 0.89) [34].

The 1000 participants were stratified into SED and more SEA groups. Participants fell into the SED group if they met any one or more of the following criteria: (a) a Parkyn Postpartum Screen score of 9 or above [30], (b) gross family income less than $20,000 per year, or (c) low social support, defined as a score below the 15^th ^percentile on the Duke-UNC Functional Social Support Questionnaire [31]. Participants whose socioeconomic status could not be determined because of missing data on the criteria listed above were excluded from the analysis.

SPSS 12.00 was used to enter and analyze the data. Descriptive statistics were used to profile the characteristics of the participants, health services needed and services used. Inferential statistics were used to compare proportions and means between the SED and more SEA groups. For categorical data 2 × 2 tables the Fisher's Exact test was used; for data with more than two categories the chi square statistic was used. The 95% confidence intervals (CI) of the proportions were computed using approximation of the normal distribution. To compare the groups with regard to continuous variables, the independent t-test was used and 95% confidence intervals around the means were calculated. The p value was set at .05. Odds ratios (OR) together with 95% confidence intervals were calculated to further evaluate differences between the SED and more SEA groups.

## Results

Of the 1000 women who entered the study at the four sites, socioeconomic status could only be determined for 726 participants due to missing data regarding family income and/or social support and/or Parkyn Postpartum Screen Scores; 295 women fell into the SED group and 431 women fell into the SEA group. The telephone interview undertaken four weeks after hospital discharge was completed by 217 of the SED women and 431 of the SEA women. The women stratified into the SED group who did not complete the telephone interview (n = 78) were compared to the SED women who completed the telephone interview (n = 217). The SED women who did not complete the telephone interview were younger (*M *= 26.6 years compared to 28.5 years, *p *< .01) and less likely to have a partner (64% compared to 79%, *p *< .025). No differences were found between the SED women who completed the telephone interview and those who did not with regard to education, language spoken at home, country of birth, baby's birth weight and having a family doctor.

For the participants who completed the study the mean age of women in the SED (*n *= 217) and more SEA groups (*n *= 431) were 28.5 years (range 16 to 43) and 30.4 years (range 19 to 42) respectively (p < .001). Forty percent of the women in the SED group and 47% of the women in the SEA group had just given birth to their first child [OR 0.75, 95% CI (0.54–1.04)].

Various attributes of the women in the SED and SEA groups are shown in Table 1. The women in the SED group were significantly less likely to have a partner, to speak English at home, to identify their ethnicity as Canadian, to have a high school diploma and were significantly more likely to have been born outside of Canada. For the women born abroad, length of time in Canada was found to be significantly associated with socioeconomic status. SED women born abroad had lived in Canada for fewer years than SEA women born abroad [*M *= 7.5 years, *SD *= 6.87; 95% CI (6.21–8.7) and *M *= 12.1 years, *SD *= 9.2; 95% CI (10.7–13.4) respectively, *p *< .001].

**Table 1:** Profile of SED and SEA postpartum women by selected attributes

**Attributes**	SED Group		SEA Group		Unadjusted
	*N^a^*	%	*N^a^*	%	OR (95% CI)
**SOCIODEMOGRAPHIC**					
Marital Status	213		424		
Has partner		79		99	0.05 (0.02–0.13)
Education	212		432		
Less than high school		25		5	2.25 (1.93–2.63)
Cultural Identity	211		428		
Canadian		50		61	0.61 (0.44–0.85)
Language Spoken at Home	217		431		
English		63		82	0.37 (0.26–0.49)
Country of Birth	215		421		
Canada		45		70	0.35 (0.25–0.49)
**WOMAN'S HEALTH**					
Overall Health	215		431		
Very good/Excellent		41		59	0.48 (0.35–0.67)
Symptoms of Postpartum Depression	211		426		
EPDS ≥ 12		19		8	2.7 (1.64–4.4)
Hospital Readmission	216		431		
YES		3		2	1.57 (0.57–4.28)
**PHYSICIAN**					
Has Family Doctor	215		430		
YES		96		97	0.74 (0.3–1.84)

Note: ^a ^Sample sizes for some variables differ from study N because of missing data. *Number who initiated breastfeeding. SED = socioeconomically disadvantaged, SEA = more socioeconomically advantaged. EPDS = Edinburgh Postpartum Depression Scale

At four weeks post discharge SED women were significantly less likely to rate their health as very good or excellent and had 2.7 times higher odds of experiencing symptoms of postpartum depression than the SEA women. Over 95% of women in both groups had family doctors. There was no significant difference between the SED and SEA groups' reported offer of the government mandated 60 hour postpartum hospital stay (48% versus 53% respectively). A higher proportion of women in the SED group (27%) reported being discharged from hospital within 24 hours after giving birth compared to the SEA group (20%) [OR 1.49, 95% CI (1.01–2.18)]. For those women who were discharged within 24 hours, there was no difference between the SED and SEA groups with regard to whether this was their first live birth (30% compared to 31% respectively, *p *< 1.0). A lower proportion of the SED group (88%) initiated breastfeeding compared to the SEA group (93%) [OR 0.53, 95% CI (0.03–0.91)]. However, at four weeks post discharge 83% of SED women and 81% of SEA women who had initiated breastfeeding were still breastfeeding [OR 0.99, 95% CI (0.65–1.53)]. Hospital readmission rates were low for both groups.

As shown in Table 2, there were no statistically significant differences between SED and SEA groups with regard to need for care for physical health problems, emotional/mental health problems, household help, reassurance/support or help with breastfeeding. Not surprisingly significantly more of the SED women needed financial aid than SEA women. A high proportion of women in SED and SEA groups reported getting help for physical health problems and breastfeeding. Although not statistically significant, a smaller proportion of the women in the SED group reported receipt of help for emotional/mental health problems. Nonetheless, approximately one third of women in both groups went without needed help for this type of problem. Similarly, only 64% of the SED group and 60% of the more SEA group who needed financial assistance received this assistance. A significantly smaller proportion of the SED women who reported needing reassurance and support were able to obtain this type of help.

**Table 2:** Postpartum women's need for and receipt of help and/or care in the first 4 weeks

Variables	SED Group			SEA Group			Unadjusted
	*N*	*%*	*n*	*N*	*%*	*n*	OR (95% CI)
Care for a physical health problem							
Needed	217	19	41	431	25	108	0.70 (0.47–1.1)
Received	41*	90	37	108*	94	102	0.54 (0.15–2.0)
Care for an emotional/mental health problem							
Needed	217	8	17	431	5	21	1.78 (0.93–3.41)
Received	17*	67	12	21*	71	15	0.80 (0.21–3.13)
Help with breast feeding^a^							
Needed	187	37	69	402	44	172	0.78 (0.54–1.10)
Received	69*	91	63	172*	93	160	0.80 (0.29–2.20)
Household help							
Needed	217	26	56	431	26	112	0.97 (0.67–1.40)
Received	56*	77	42	112*	90	101	0.43 (0.17–1.10)
Reassurance/support							
Needed	217	25	54	431	27	115	0.89 (0.61–1.30)
Received	54*	85	44	115*	98	113	0.11 (0.02–0.48)
Financial Support							
Needed	217	18	38	431	3	10	7.08 (3.69–13.6)
Received	36*	64	24	10*	60	6	1.05 (0.33–3.25)

Note: ^a ^Only women who initiated breastfeeding are included.* Number reported needing the service. SED = socioeconomically disadvantaged, SEA = more socioeconomically advantaged

No statistically significant differences in use of physician and midwifery services were found between groups (Table 3). Family doctors were the most frequently accessed primary health care providers. Women in both groups made up to four visits, with 75% of the SED group and 73% of the SEA group having made only one visit. Obstetrician-gynaecologists were the second most reported physician service used by both groups. Few women reported using emergency rooms and walk-in clinics.

**Table 3:** Postpartum women's utilization of physician and midwifery services

Physician and Midwifery Services	SED Group (*N *= 217)		SEA Group (*N *= 431)		Unadjusted
	*%*	*n*	*%*	*n*	OR (95% CI)
Visit to family physician	30	65	28	121	1.06 (0.84–1.34)
Phone call to family physician	9	20	8	35	1.09 (0.76–1.58)
Visit to midwife	2	4	4	17	0.64 (0.95–1.48)
Visit from midwife	4	9	7	30	0.72 (0.40–1.28)
Phone call to midwife	1	2	2	9	0.74 (0.28–1.99)
Visit to an OBGYN	12	26	8	35	1.33 (0.98–1.82)
Phone call to OBGYN	7	15	6	26	1.04 (0.69–1.57)
Visit to another specialist	2	4	3	13	0.93 (0.46–1.94)
Emergency room visit	5	11	4	17	1.10 (0.68–1.78)
Walk-in clinic visit	2	4	4	17	0.70 (0.33–1.53)

Note: SED = socioeconomically disadvantaged, SEA = more socioeconomically advantaged

Over 80% of women in both groups reported having received a telephone call from a public health nurse (Table 4). However the SED group was significantly less likely to receive the phone call within the government mandated 48 hours after hospital discharge. A high proportion of women in both groups were offered a home visit. The odds of accepting the offer of a public health nurse home visit were more than two times higher in the SED group. In addition the SED group's odds for continued involvement with public health were 2.5 times higher at four weeks post hospital discharge than the SEA group. The SED group received significantly more public health nurse home visits (*p *< .001).

**Table 4:** Use of public health nursing (PHN) services in the first 4 weeks after leaving hospital

Type of contact	SED Group			SEA Group			Unadjusted
	*N*	*%*	*n*	*N*	*%*	*n*	OR (95% CI)
Received a PHN phone call	217			431			
Yes		81	176		86	371	0.69 (0.45–1.07)
PHN phone call within 48 hours after discharge?	158^a^			371^a^			
Yes		87	137		98	366	0.31 (0.13–0.77)
PHN home visit offered in phone call	176^a^			366^a^			
Yes		95	167		94	347	1.12 (0.53–2.63)
PHN home visit accepted	167*			347*			
Yes		77	128		60	208	2.24 (1.47–3.40)
Continued PHN services at 4 weeks post discharge	124*			206*			
Yes		34	42		17	35	2.5 (1.49–4.21)

	SED Group			SEA Group			
	*N*	*M*	SD 95% CI	*N*	*M*	SD 95% CI	*P*

Age in days of baby at the first PHN home visit?	124*	6.9	5.5 (5.8–7.7)	206*	7.7	6.2 (7.0–10.1)	p < 0.23
Number of PHN home visits	123*	1.9	1.5 (1.6–2.2)	207*	1.4	0.76 (1.2–1.5)	p < 0.001

Note: ^a ^Number that received a PHN phone call who answered the question.

*Number offered a PHN home visit who answered the question. SED = socioeconomically disadvantaged, SEA = more socioeconomically advantaged

Table 5 compares the proportion of women in the SED and SEA groups who used other community based health and social services. Breastfeeding clinics were the service most often used by both groups. The SED group was significantly more likely to use food banks, social assistance, and the Children's Aid Society. Few women in both groups reported using services such as postpartum support groups, parenting classes, and family resource centres in the first four weeks at home.

**Table 5:** Health and social services use by SED and SEA groups

Health Provider or Social Services	SED Group (N = 217)		SEA Group (N = 431)		Unadjusted
	*%*	*n*	*%*	*n*	OR (95% CI)
**Health Providers**					
Hospital/clinic nurse	8	17	7	30	1.09 (0.73–1.62)
Lay home visitor	3	6	1	4	2.03 (1.26–3.26)
Telephone nurse	7	15	12	52	0.67 (0.42–1.05)
Other care provider	7	15	8	34	0.98 (0.64–1.49)
**Services**					
Parenting class	3	6	3	13	0.98 (0.51–1.93)
Postpartum support group	1	2	1	4	1.29 (0.54–3.05)
Family resource centre	4	8	2	9	1.52 (0.94–2.44)
Parenting/child drop-in	7	15	3	13	1.53 (1.06–2.25)
Children's Aid Society	4	8	0	0	3.08 (2.75–3.43)
Canadian Prenatal Nutrition Program	1	2	0.2	1	2.26 (1.27–4.03)
Food bank	5	11	0	0	3.07 (2.75–3.44)
Women's shelter	0.5	2	0.2	1	1.48 (0.37–6.01)
Breastfeeding clinic	13	28	18	77	0.76 (0.54–1.06)
Social assistance	9	19	0.5	2	2.87 (2.40–3.44)
Other services	5	11	5	22	1.03 (0.63–1.68)

SED = socioeconomically disadvantaged, SEA = more socioeconomically advantaged

## Discussion

This is one of the first studies to examine SED postpartum women's health and social service needs and use. Although this study did not use a random sample of women who had medically uneventful vaginal deliveries of healthy infants it provides a snapshot of SED women delivering in large Canadian urban centres and of their health and social service needs and use in the early postpartum weeks.

Many of the attributes of SED women in this study were similar to those found in previous research including that they were more likely to be single, younger, less educated and in poorer overall health [16,35]. However, distinct from previous research findings [15], a high proportion of SED women continued to breastfeed four weeks post hospital discharge.

The high representation of women born abroad (55%) in the SED group is a concern. Researchers have found that immigrant postpartum women are more likely to get sub-optimal care in hospital and in the community [36] and use health services less frequently than non immigrant women [37], particularly preventive health services [38]. The immigrant women in the SED group had lived in Canada a shorter period of time than those in the more SEA group. The findings may reflect the fact that new immigrants are often socially isolated due to separation from family and other social support networks [39] and have not had enough time to establish new social support networks. Financial hardship experienced by many new immigrants may also explain the high percentage of immigrant women in the SED group, as many new immigrants experience underemployment or unemployment [40] which likely worsens with maternity leave.

Only half of the SED women in this study reported being offered the currently mandated 60-hour postpartum hospital stay [28], indicating that the policy is not being universally implemented. One in four of the SED women were discharged from hospital within 24 hours after giving birth, in contrast to one in five of the more SEA women. Service use findings in this study indicate that these SED women were not receiving added supportive services following discharge as recommended by Canadian obstetric and pediatric guidelines on early discharge [41]. Some SED women might have opted to leave hospital early due to negative experiences with care, as negative attitudes, lack of respect, dissatisfaction with treatment, and impersonal care have been reported as barriers to health service utilization [21].

Surprisingly few differences were found between the SED and SEA groups with regard to self-reported health service needs and use. The women in the SED group may have been too busy with the multiple challenges of their daily lives, related to socioeconomic disadvantage and a new baby, to focus on their own health needs. Given the SED group's health service utilization patterns, family doctors and public health nurses may be best positioned to screen for mental and physical health problems and offer appropriate referrals and interventions.

The SED women's inability to get needed support and reassurance is a concern. Provision of support and reassurance outside of the realm of personal social support potentially comes from community services and programs such as postpartum support groups, public health nurse home visiting, and lay home visitors. Both groups reported very low use of these types of services. As almost all the women in the SEA group reported that they received help in this domain, they likely obtained this support and reassurance from informal sources such as family and friends. More research is necessary to understand the nature of support that is needed by SED postpartum women and to develop effective strategies to deliver such support.

A large proportion of women in the SED group took advantage of Ontario's public health postpartum home visiting program. The SED women reported more public health visits than the SEA women; however the difference of a half visit (mean difference of 0.522) more in the first four weeks at home is likely not clinically significant. In addition it is not known how much of this service was oriented toward promotion of the women's health. The program's focus is primarily on promoting the health of at-risk children [28]; promoting mother's health is secondary and important to the degree that it affects the health of her child. Yet, the high acceptance of a home visit by SED postpartum women may indicate that they want or need the services of a public health nurse in the early postpartum weeks. The high acceptance rates may also reflect the ability of public health nurses to encourage a visit during the phone call, especially to the SED group. There should be some concern about the 19% of SED women who did not receive a public health nurse phone call. The initial postpartum telephone call is a primary point of entry into Ontario's public health postpartum follow-up program.

The low use of community services such as parenting classes and postpartum support groups may signify a lack of interest in these types of programs in the early postpartum weeks. However the low use of these programs may also indicate the existence of barriers to accessing these types of resources, such as lack of transportation and knowledge about the services.

The generalizability of these study findings is limited by the use of a non random sample of medically low risk mothers and infants. In addition, the SED women who were excluded from the analysis differed on some characteristics from the SED women who were included. Furthermore the identification of SED women was limited by the variables measured in the larger mother-infant survey. For example, the low-income cut-off in large Ontario cities for a family of two is $25,867 [42] whereas the data did not allow for discrimination between women with family incomes ranging from $20,000 to $40,000. Finally, the use of health and social services was measured by women's self report, potentially resulting in recall bias and should be confirmed by other methods such as physician and public health chart audits.

## Conclusion

The postpartum period presents a unique window of opportunity for community-based health and social service providers and programs to contribute to the elimination of health disparities experienced by SED women and in turn their children. At government and community agency levels, policy makers must recognize that SED women are a heterogeneous group who are at high risk for experiencing health inequities in the postpartum period. Consequently, resources need to be dedicated, and a spectrum of innovative approaches must be developed to support effective health and social services that focus on both the health of these women and their infants. The findings indicate that public health nurses and family physicians are well situated to assist SED women in accessing needed services in the early postpartum weeks. More research is required to further examine what kinds of services SED women need in the early postpartum weeks, and what facilitates and hinders access to these services. Phase II of our mixed methods study takes a qualitative descriptive approach to further investigate SED postpartum women's experiences in the first four weeks at home to understand their health service needs and the spectrum of facilitators and barriers to accessing health services.

## Competing interests

The authors declare that they have no competing interests.

## Authors' contributions

CKL conceived of and designed the study, undertook the statistical analysis, interpretation and drafted the manuscript. WS contributed to the study design, interpretation of the results and critically reviewed the manuscript. DC contributed to the study design, interpretation of results and critically reviewed the manuscript. All authors read and approved the final manuscript.

## Pre-publication history

The pre-publication history for this paper can be accessed here:


